# Fluid balance and renal outcomes in patients requiring renal replacement therapy in the ICU

**DOI:** 10.1186/cc12372

**Published:** 2013-03-19

**Authors:** JA Silversides, R Kuint, R Pinto, R Wald, M Hladunewich, NK Adhikari

**Affiliations:** 1 University of Toronto, Canada

## Introduction

Fluid overload is associated with mortality in critically ill patients with acute kidney injury (AKI) [1-3]. We explored this relationship in patients with AKI who received renal replacement therapy (RRT) in the ICU to investigate the relationship between fluid balance and intradialytic hypotension with mortality and recovery of renal function.

## Methods

We conducted a retrospective cohort study among patients aged ≥16 years who had RRT initiated and continued for ≥2 days in a level 2 or 3 ICU at two academic centres, and had fluid balance data available. Patients with end-stage kidney disease, within 1 year of a renal transplant or who had RRT initiated to treat a toxic ingestion were excluded. We used multivariable logistic regression to determine the relationship between mean daily fluid balance over the first 7 days following RRT initiation and the outcomes of mortality and RRT dependence in survivors.

## Results

A total of 522 patients were included (319 male, mean age 64 years); 264 (50.6%) died in hospital. Survivors and those who died were similar with respect to age, weight and incidence of heart failure, liver cirrhosis and primary renal diagnoses. Independent risk factors for hospital mortality were higher Sequential Organ Failure Assessment score at RRT initiation (odds ratio (OR) = 1.18, 95% confidence interval = 1.11 to 1.25), higher Charlson comorbidity index (OR = 1.22, 1.09 to 1.37), lower baseline creatinine (OR = 0.984 per 10 μmol/l, 0.970 to 0.998), lower minimum mean arterial pressure (MAP) on day 1 (OR = 0.81 per 10 mmHg, 0.66 to 0.95) and more positive fluid balance for the first 7 days (OR 1.03 per 100 ml, 1.02 to 1.05). Of 172 hospital survivors, 57 (33.1%) were RRT dependent at discharge. Although univariable analysis suggested lower SOFA scores and higher baseline serum creatinine levels in those who remained RRT dependent, no factor was independently associated with RRT dependence at discharge in a multivariate model.

## Conclusion

In this cohort of patients with AKI requiring RRT, a more positive fluid balance over 1 week and lower initial minimum MAP were associated with mortality. Among survivors, a less positive fluid balance was not associated with increased risk of RRT dependence at discharge, suggesting that conservative fluid management does not significantly attenuate renal recovery.
